# Efficacy of Intraosseous Versus Intravenous Drug Administration in Out-of-Hospital Cardiac Arrest: A Systematic Review and Meta-Analysis

**DOI:** 10.7759/cureus.72276

**Published:** 2024-10-24

**Authors:** Godfrey Tabowei, Samuel K Dadzie, Ashique Ali Khoso, Abdallah A Riyalat, Muhammad Ali, Mohamed Ismael Mohamed Samir Ismael Atta, Calvin R Wei, Neelum Ali

**Affiliations:** 1 Internal Medicine, Texas Tech University Health Sciences Center, Odessa, USA; 2 Internal Medicine, Piedmont Athens Regional Medical Center, Athens, USA; 3 Cardiovascular Medicine, Pir Abdul Qadir Shah Jeelani Institute of Medical Sciences, Gambat, PAK; 4 Internal Medicine, Sidra Medicine, Doha, QAT; 5 Cardiology, Rawalpindi Institute of Cardiology, Rawalpindi, PAK; 6 Internal Medicine/Pediatrics, Faculty of Medicine, Alexandria University, Alexandria, EGY; 7 Research and Development, Shing Huei Group, Taipei, TWN; 8 Internal Medicine, University of Health Sciences, Lahore, PAK

**Keywords:** intraosseous access, intravenous access, meta-analysis, out-of-hospital cardiac arrest, resuscitation

## Abstract

This meta-analysis compared the efficacy of intraosseous (IO) versus intravenous (IV) drug administration in out-of-hospital cardiac arrest (OHCA). We systematically searched Embase, Web of Science, PubMed, and Cochrane Library through September 20, 2024, for relevant studies. The primary outcome was favorable neurological outcome, with secondary outcomes, including survival to hospital discharge and return of spontaneous circulation (ROSC). Seventeen studies, including randomized controlled trials and observational studies, were included in the final analysis. Pooled results showed that IV access was associated with significantly better outcomes compared to IO access. Patients in the IV group had 1.73 times higher odds of favorable neurological outcomes (RR: 1.73, 95% CI: 1.32-2.27), 1.64 times higher odds of survival to hospital discharge (RR: 1.64, 95% CI: 1.27-2.12), and 1.27 times higher odds of ROSC (RR: 1.27, 95% CI: 1.16-1.40). However, significant heterogeneity was observed across studies for all outcomes. These findings suggest that IV access may be superior to IO access in improving outcomes for OHCA patients. However, the high heterogeneity and conflicting results from individual studies highlight the need for careful interpretation and further research. Factors such as ease of access, speed of establishment, and patient condition should also be considered when choosing between IV and IO routes during resuscitation. This meta-analysis underscores the importance of reassessing current guidelines and conducting more robust primary studies to optimize vascular access strategies in OHCA management.

## Introduction and background

Out-of-hospital cardiac arrest (OHCA) remains a critical public health challenge, with survival rates being alarmingly low despite advancements in emergency medical services (EMS) and resuscitation techniques [1,2]. Immediate and effective intervention is paramount in improving the chances of survival and favorable neurological outcomes. One of the most crucial steps in the resuscitation process is the administration of drugs, including vasopressors, antiarrhythmics, and fluids [3,4]. The success of drug delivery, however, is contingent upon timely and reliable vascular access, which in the chaotic environment of OHCA can be difficult to achieve [5].

Traditionally, intravenous (IV) access has been the gold standard for drug administration during resuscitation. It allows for the rapid infusion of medications directly into the bloodstream, offering quick pharmacologic action [6]. However, IV access can be time-consuming, especially in emergency situations where peripheral veins may collapse due to hypoperfusion or shock [7]. In such cases, delays in obtaining IV access could negatively impact patient outcomes. Intraosseous (IO) access, which involves injecting drugs directly into the bone marrow, has emerged as a viable alternative [8]. IO access can be established more quickly and is less technically demanding than IV access in patients with difficult vascular conditions. Moreover, IO access is believed to provide rapid drug delivery to the central circulation, which is critical during the resuscitation process [9].

Despite these advantages, the efficacy of IO access in improving survival outcomes compared to IV access remains a subject of debate. Several studies have explored the pharmacokinetics, ease of use, and success rates of IO versus IV access in various resuscitation scenarios, including OHCA [10-12]. However, conflicting results have been reported, and there is limited consensus on which method should be the preferred choice during resuscitation.

Given the clinical relevance of this issue, a comprehensive evaluation of the available evidence is necessary to formulate guidelines and improve resuscitation protocols. This meta-analysis aims to systematically compare the efficacy of IO versus IV drug resuscitation in OHCA, focusing on key outcomes such as return of spontaneous circulation (ROSC), survival to hospital admission, survival to discharge, and neurological outcomes. By synthesizing the existing literature, this study seeks to provide clarity on the optimal approach to vascular access in the context of OHCA, offering valuable insights for both clinical practice and future research.

## Review

Methods 

The Preferred Reporting Items for Systematic Reviews and Meta-Analyses (PRISMA) standards were followed in this review.

Literature Search 

Two authors independently searched for online databases, including Embase, Web of Science, PubMed, and Cochrane Library from 2017 to 20 September 2024. Keywords used for searching relevant articles included “Intraosseous”, “intravenous”, and “out-of-hospital cardiac arrest”. No restrictions were placed on language and year of publication. In order to make sure that no relevant article is left out, relevant review articles and bibliography lists of included articles were also manually screened. In case of disagreements, consensus was made through discussion.

Study Selection 

Two authors scanned the abstracts and titles of all articles obtained through database searching to determine whether the articles were relevant to include in this meta-analysis. The following inclusion criteria were used: (a) individuals with OHCA, (b) comparison between IV access and IO access for drug administration during cardiopulmonary resuscitation (CPR), (c) outcomes assessed including favorable neurological outcomes (as defined by individual studies), survival till discharge and return of spontaneous circulation (ROSC), and (d) randomized control trials (RCT) and non-RCTs. We excluded single-arm studies, reviews, case series, editorials, and non-human studies. We also excluded studies that included patients other than OHCA. Full-text articles were retrieved after initial screening, and a detailed investigation was done. In case of disagreements, consensus was made through discussion.

Data Extraction and Quality Assessment 

In this meta-analysis, the primary outcome was a favorable neurological outcome. Secondary outcomes included survival at hospital discharge and ROSC. Two authors extracted quantitative data using a data extraction form developed on Microsoft Excel (Microsoft® Corp., Redmond, WA). Data extraction was developed by the principal author, and it was cross-checked by the second author before using for data extraction. Any disagreement occurring during this stage was resolved through discussion. Data extracted from included studies were author name, year, design, sample size, and outcomes (including unadjusted or adjusted measures). Quality assessment was performed by two authors independently using the Newcastle-Ottawa scale and Cochrane Risk of Bias tool for observational studies and RCTs, respectively.

Data Analysis 

We performed data analysis using RevMan (version 5.4.1; Cochrane Collaboration, London, UK) and took risk ratio (RR) as the effect estimate for the synthesis of data, along with a 95% confidence interval (CI). The P-value for significance was kept at 0.05. We used a fixed or random effect model based on the heterogeneity among the study results. Heterogeneity was calculated as I-square, where an I-square value of >50% showed significant heterogeneity.

Results 

**Figure 1:**
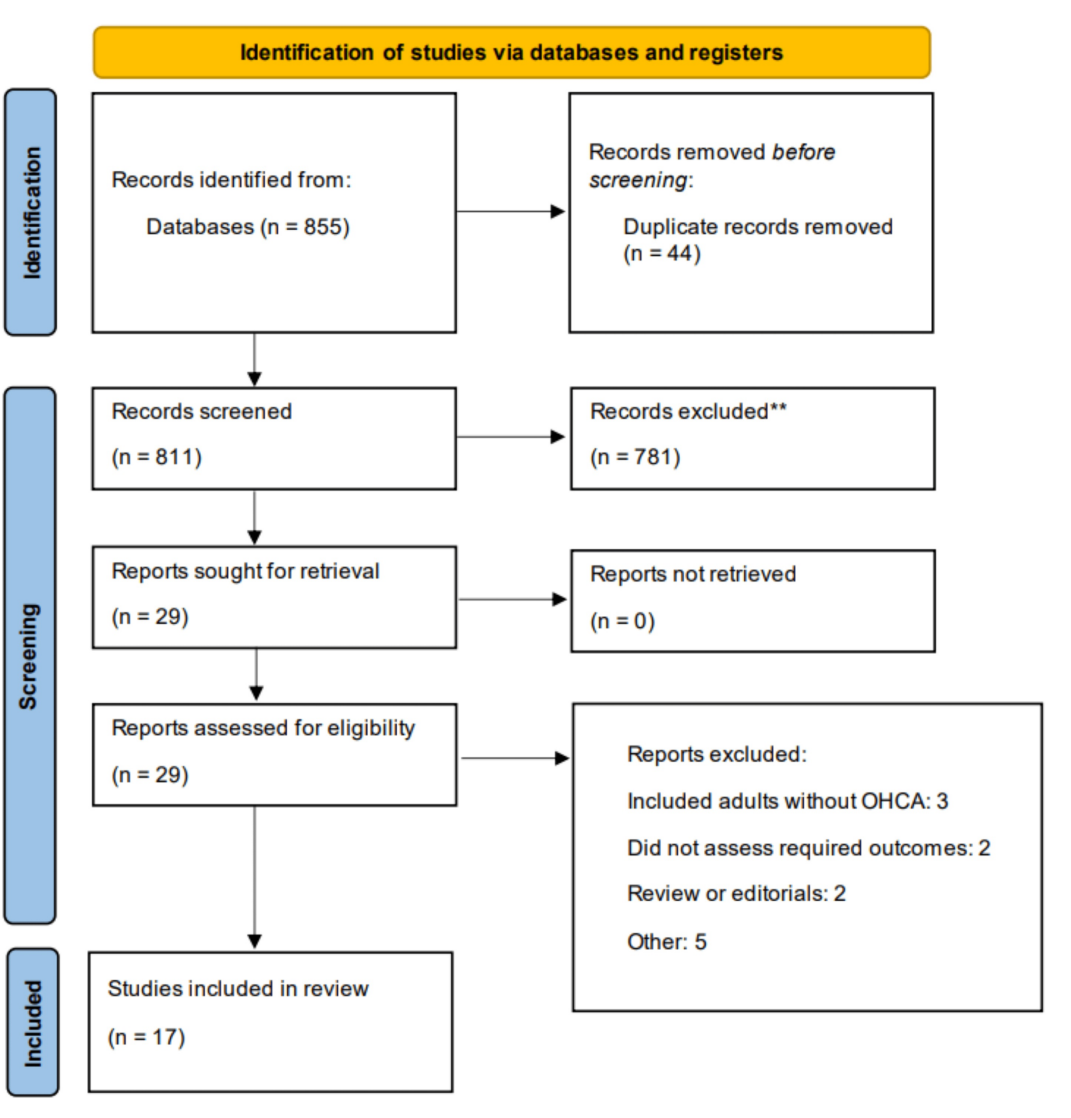
PRISMA flowchart (study selection process) PRISMA, Preferred Reporting Items for Systematic Reviews and Meta-Analyses

Through electronic searching, we identified 855 studies. After removing 44 duplicates, an initial screening of 811 records was done. A detailed assessment of 29 articles was done. Finally, 17 studies were included in this meta-analysis. Figure 1 shows the process of study selection. Table 1 presents the characteristics of the included studies. All of the studies were published between 2018 and 2024. Out of 17 studies, 11 were observational, two were randomized-control trials (RCTs), and four were secondary analyses of RCTs.

**Table 1:** Study characteristics IV: Intravenous; IO: Intraosseous; RCT: Randomized-control trial; NR: Not reported

Author ID	Study Design	Groups	Sample Size	Route of IO	Mean Age (Years)	Male (n)	Initial Shockable Rhythm (n)	Quality Assessment Results
Baert et al., 2020 [13]	Retrospective	IV	27280	Humeral/Tibial	67	19207	NR	High
		IO	1576		64	975		
Benner et al., 2023 [14]	RCT (Secondary analysis)	IV	822	Humeral	67	589	NR	Moderate
		IO	1000		64	666		
Brebner et al., 2024 [15]	Retrospective	IV	1575	Humeral	66	1147	310	High
		IO	537		63	379	67	
Clemency et al., 2017 [11]	Retrospective	IV	778	NR	63	515	111	Moderate
		IO	552		59.8	339	90	
Daya et al., 2020 [16]	RCT	IV	2347	Tibial	62.7	1935	NR	Moderate
		IO	657		62.3	481		
Feinstein et al., 2017 [9]	Retrospective	IV	1525	Tibial	64.3	984	454	High
		IO	275		61.5	142	47	
Hamam et al., 2021 [17]	Retrospective	IV	4293	Tibial	64.5	2694	832	High
		IO	2603		65.2	1448	325	
Kawano et al., 2018 [12]	RCT (Secondary analysis)	IV	558	NR	64	295	73	Moderate
		IO	558		64	297	83	
Ko et al., 2024 [18]	RCT	IV	991	Humeral/Tibial	66	713	285	Moderate
		IO	741		64	521	217	
Lee et al., 2024 [10]	Retrospective	IV	1602	Humeral/Tibial	69	1088	325	Low
		IO	401		71	246	67	
Mody et al., 2019 [19]	RCT (Secondary analysis)	IV	16663	Tibial	68	11064	3882	Low
		IO	3068		65	1743	534	
Monaco et al., 2023 [20]	Retrospective	IV	29,688	Humeral/Tibial	NR	19802	7511	Low
		IO	1303			812	197	
Nguyen et al., 2019 [21]	Retrospective	IV	353	Tibial	64.7	292	103	Moderate
		IO	342		66	202	62	
Nilsson et al., 2023 [22]	Retrospective	IV	5979	Humeral/Tibial	70	3956	NR	Low
		IO	773		67	503		
Nolan et al., 2020 [23]	RCT (Secondary analysis)	IV	2354	Humeral/Tibial	70.9	3364	1031	Moderate
		IO	1063		67.5	1355	318	
Vadeyar et al., 2023 [24]	Retrospective	IV	43660	NR	72.6	28657	11,125	Low
		IO	18288		68.9	11270	2853	
Zhang et al., 2020 [25]	Retrospective	IV	27758	NR	65	3366	1006	Low
		IO	7975		65	3369	977	

Meta-analysis of Outcomes 

**Figure 2:**
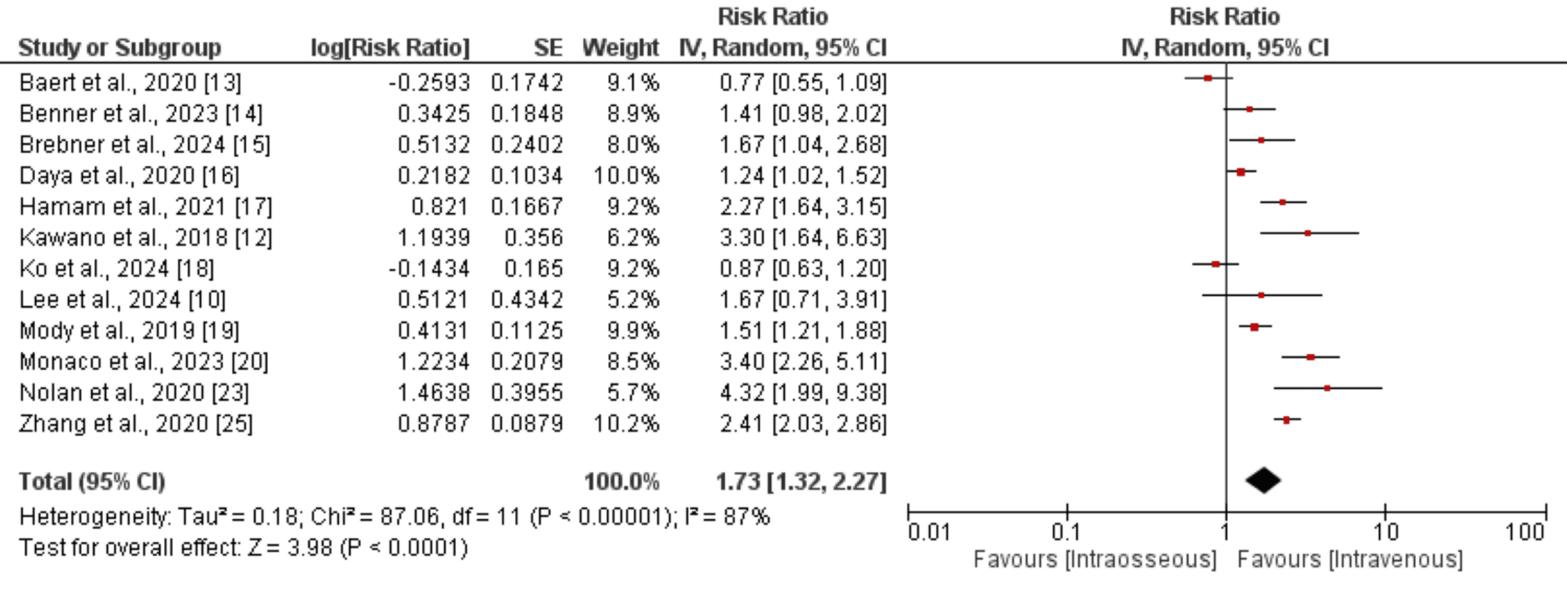
Comparison of favorable neurological outcomes between the two groups References [10,12-20,23,25]

Favorable neurologic outcomes: A pooled analysis of 12 studies was conducted to determine the incidence of favorable neurological outcomes between IV and IO access. The results are presented in Figure 2. As shown in Figure 2, the incidence of favorable neurological outcomes is 1.73 times greater in patients in the IV group compared to the IO group (RR: 1.73, 95% CI: 1.32-2.27). The I-square value of pooled analysis is 87%, showing high heterogeneity among the study results.

**Figure 3:**
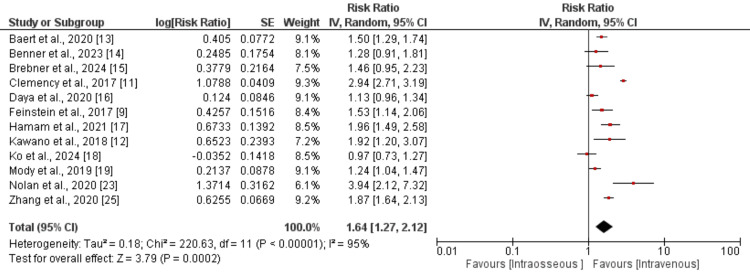
Comparison of survival to hospital discharge between the two groups References [9,11-19,23,25]

Survival to hospital discharge: A pooled analysis of 12 studies was performed to determine the survival at hospital discharge between IV and IO. The results are presented in Figure 3. As shown in Figure 3, the overall survival was significantly higher in patients in the IV group compared to patients in the IO group (RR: 1.64, 95% CI: 1.27-2.12). The I-square value of pooled analysis is 95%, showing high heterogeneity among the study results.

**Figure 4:**
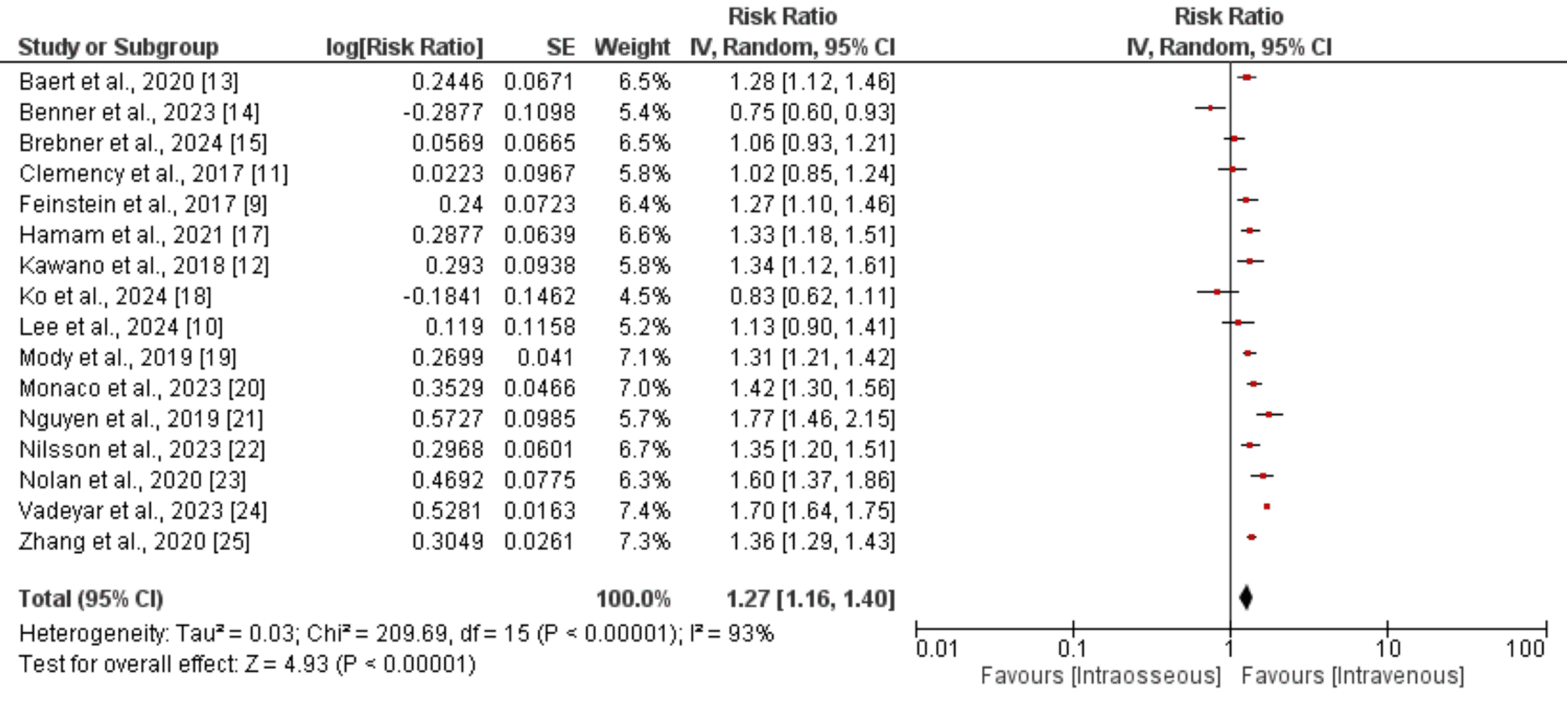
Comparison of ROSC between the two groups ROSC, Return of spontaneous circulation References [9-15,17-25]

ROSC: A pooled analysis of 13 studies was performed to determine the ROSC between IV and IO. The results are presented in Figure 4. As shown in Figure 4, ROSC was significantly higher in patients in the IV group compared to patients in the IO group (RR: 1.27, 95% CI: 1.16-1.40). The I-square value of pooled analysis is 93%, showing high heterogeneity among the study results.

Discussion 

The aim of this meta-analysis is to compare the outcomes between IV and IO access in patients with OHCA. The following is the summary of the findings of this study: (a) the incidence of favorable neurological outcomes was significantly higher in patients in the IV group; (b) the overall survival was significantly higher in patients in the IV group compared to patients in the IO group; and (c) ROSC was significantly higher in patients in the IV group compared to patients in the IO group. The findings of this study support the use of IV compared to IO.

Two meta-analyses in the past showed that IO access was related to poor outcomes of OHCA compared to IV access [26,27]. The findings of this meta-analysis also showed poor outcomes in IO access compared to IV access. Compared to this meta-analysis, our review included recently conducted studies, such as observational studies and RCTs. However, as Granfeldt et al. [27] pointed out, our heterogeneity was still quite considerable, requiring a careful interpretation of our study and additional research into the heterogeneity's causes.

Moving on to the present review, our analysis revealed that there is still inconsistency in the evidence about how IO access affects resuscitation patient outcomes. For example, some investigations reported no significant difference between IO and IV routes, while others suggested that IO access would be associated with reduced rates of ROSC or worse survival outcomes upon hospital discharge [28]. Another study, however, suggested that short-term survival and IO access were inversely related [25]. The need for more thorough primary research is highlighted by these conflicting results, which may be related to the original studies' differing methodological quality and evidence dependability. When considering the best access technique for resuscitation, the success rate refers to how effectively the chosen method, such as peripheral IV or IO access, can be established, especially in time-critical situations. The success rate of securing access is influenced by various factors, including patient characteristics, underlying medical conditions, and the urgency of the situation. For peripheral IV access, conditions such as severe dehydration, hypovolemia, or shock can cause veins to collapse, making IV insertion more challenging. Additionally, factors such as obesity, which can obscure veins, and the presence of edema, which may make veins difficult to visualize or palpate, can further reduce the likelihood of successful IV placement. These factors must be weighed alongside other considerations such as the speed of access establishment, potential complications, and the overall success rate when choosing between IV and IO access in emergency settings [27]. In contrast to IV cannulation, IO access is renowned for its quick placement and comparatively greater success rates, particularly in circumstances when obtaining IV access may be challenging. Despite the contradictory data about IO access's effect on patient outcomes, this can make it a more appealing choice in some situations [27].

We included a recent RCT that found that, although the differences were not statistically significant, a greater percentage of patients had a pre-hospital ROSC following IO access. In the per-protocol analysis of this study, patients who achieved successful IO access initially exhibited a significantly lower rate of sustained ROSC and tended towards worse outcomes compared to those with IV prior to adjustment with confounding variables. However, after adjustment, the outcomes shifted. In the comparison of the two groups, those who successfully obtained IV access had a higher frequency of witnessed arrests and shockable rhythms, which are associated with improved outcomes. This result could be attributed to the notion that IO access success is less influenced by the patient’s condition, while selecting the IV route is more specific. The potential mechanism underlying the comparable effects observed between the two methods in our study implies that patients with IV access are in the poorest condition [18].

Furthermore, even while the AHA guidelines now advise IO access in cases where IV attempts fail or are impractical, the recommendation's strength (COR 2b, LOE B-NR) implies that the evidence supporting it is weak or contradictory [29]. Our results thus confirmed the necessity for additional study to examine how IO access affects resuscitation results and to ascertain whether the present guidelines need to be updated.

Study limitations

Our study has certain limitations. First, we lacked sufficient data to incorporate the IO insertion site into the subgroup analysis. Compared to tibial IO access, humeral IO access for adrenaline administration has been demonstrated to reach a higher maximum serum concentration in a shorter amount of time, increasing the likelihood of survival. The significant heterogeneity might have been partially explained by variations in the choice of IO sites. Second, we did not examine combinations of all possible effect estimates; instead, we abstracted one representative effect estimate for synthesis in the meta-analysis for each trial. However, we believed that type I mistake could likely be avoided by using a hypothesis-driven meta-analysis with a predetermined abstraction process. Lastly, while all of the included research classified neurological outcomes using recognized evaluation systems, the majority of these studies evaluated the outcomes after the fact, which could introduce misclassification bias and provide non-significant results.

## Conclusions

This meta-analysis suggests that IV access may be associated with better outcomes than IO access in OHCA resuscitation. Patients receiving IV drug administration demonstrated higher rates of favorable neurological outcomes, survival to hospital discharge, and ROSC. However, the significant heterogeneity among studies necessitates a cautious interpretation of these findings. While our results support the preferential use of IV access when feasible, the established benefits of IO access, such as ease and time taken to achieve success, should not be overlooked in challenging clinical scenarios. Further high-quality research is needed to elucidate the factors influencing the efficacy of these access routes and to optimize their use in various resuscitation contexts. Future studies should also consider the impact of IO insertion sites and other variables on patient outcomes.
